# Innovative and Healthy Cookies Enriched with Blueberry Leaf Powder

**DOI:** 10.3390/molecules30183671

**Published:** 2025-09-09

**Authors:** Francesco Antonio Santuccione, Marina Soazo, Emilce Llopart, Matías Rossi, Roxana Andrea Verdini, Paola Pittia, Leonardo Martín Pérez

**Affiliations:** 1 Facultad de Ciencias Bioquímicas y Farmacéuticas, Universidad Nacional de Rosario, Suipacha 531, Rosario S2002LRK, Argentina; fasantuccione@gmail.com (F.A.S.); soazo@iquir-conicet.gov.ar (M.S.); llopart@iqui-conicet.gov.ar (E.L.); mrossi@fbioyf.unr.edu.ar (M.R.); verdini@iquir-conicet.gov.ar (R.A.V.); 2 Department of Biosciences and Technology for Food, Agriculture and Environment, University of Teramo, Via R. Balzarini 1, 64100 Teramo, Italy; 3 Consejo Nacional de Investigaciones Científicas y Técnicas (IQUIR-CONICET, UNR), Suipacha 531, Rosario S2002LRK, Argentina; 4 Facultad de Química e Ingeniería del Rosario, Pontificia Universidad Católica Argentina, Av. Pellegrini 3314, Rosario S2002QEO, Argentina

**Keywords:** functional foods, bakery products, agricultural by-products, antioxidants, dietary fiber

## Abstract

Blueberry (*Vaccinium* spp.) leaves, a residual biomass of pruning, are a rich source of polyphenols, fiber, and minerals. In this study, blueberry leaf powder (BBLP) was incorporated into wheat/soy flour-based cookies to develop antioxidant- and fiber-enriched bakery products. BBLP exhibited 8.2% protein, 44% dietary fiber (predominantly insoluble), and high antioxidant activity (2109 ± 20 mg gallic acid equivalents/100 g sample; 6251 ± 42 µmol Trolox equivalents/100 g). Four cookies’ formulations were prepared by replacing 0%, 2.5%, 5.0%, and 7.5% of the flour blend with BBLP. The total phenolic content, total antioxidant content, physical properties (weight, diameter, thickness, volume, hardness, and color), chemical composition (moisture, ash, minerals, protein, carbohydrate, fat, and fiber content), and sensory properties (taste, texture, aroma, and overall acceptability) were analyzed. All BBLP-enriched cookies qualified as a “source of fiber” according to Codex Alimentarius guidelines and EU Regulation (EC) No 1924/2006 on nutrition and health claims for foods. The addition of BBLP significantly affected the cookies’ diameter, thickness, volume, and hardness, likely due to its high insoluble fiber content. Moreover, as BBLP levels increased, the surface color darkened progressively, with increased redness and decreased yellowness attributed to the presence of anthocyanins. Accordingly, BBLP-enriched cookies showed increased antioxidant capacity, proportional to the amount of BBLP added, indicating good retention of the bioactive compounds after baking. Sensory evaluation using Quantitative Descriptive Analysis revealed that cookies with 2.5% BBLP were rated with the highest acceptability scores, whereas higher concentrations imparted noticeable herbal notes and a darker color, decreasing overall acceptability. In conclusion, BBLP can be effectively incorporated at 2.5% to enhance the nutritional quality and antioxidant potential of cookies without compromising sensory appeal, contributing to sustainable food innovation by valorizing residual agricultural biomass.

## 1. Introduction

Blueberries (*Vaccinium* spp.) have gained increasing attention not only for their sensory and nutritional attributes but also for their economic importance. Global production has expanded rapidly in the last decade, exceeding 1.8 million metric tons in 2021, driven by both consumer demand and the development of new cultivars, positioning the fruit as a leading commodity in the food market [1]. This growth has led to a significant increase in by-products from blueberry production, particularly leaf residues generated through pruning operations. Although no specific data on leaves produced during routine harvesting are available, they represent a significant and abundant residual material. If left unmanaged, this biomass can contribute to environmental burdens such as greenhouse gas emissions during decomposition and nutrient runoff, and may also incur disposal costs, representing a lost economic and material resource considering the inputs used for cultivation [2].

Although often discarded or underutilized, blueberry leaves are rich in polyphenols, flavonoids, and phenolic acids, with demonstrated biological activity and potential health-promoting properties [3]. Recent studies have reported that the leaves contain even higher concentrations of bioactive compounds than the fruit itself, including chlorogenic acid and other caffeoylquinic acids, quercetin and kaempferol glycosides, catechins, proanthocyanidins, anthocyanins, triterpenoids (e.g., ursolic and oleanolic acids), and phytosterols [4,5,6,7]. These constituents are associated with a broad spectrum of biological effects, including antioxidant, anti-inflammatory, antiviral, and cardiometabolic benefits [8,9]. Compared with other berry leaves such as bilberry, blackberry, and raspberry, blueberry leaves exhibit a predominance of caffeoylquinic acids and rutin derivatives, distinguishing them as a particularly valuable functional ingredient, although reported concentrations vary considerably depending on the cultivar, growing conditions, and the extraction and quantification methods employed [10,11,12]. Consequently, increasing efforts are being directed toward their revalorization in the food chain [9], in alignment with sustainable development goals and circular bioeconomy principles.

Dried blueberry leaves have traditionally been used in herbal teas due to their chemical richness and mild flavor, which has contributed to their popularity as a natural infusion in various cultures to promote health and wellness. In addition to phenolics, blueberry leaves also provide significant amounts of insoluble dietary fiber and protein, enhancing their nutraceutical potential [13]. While most studies have focused on extracts or infusions, the promising bioactive profile of the whole leaf matrix underscores its broader applicability in food formulations [3]. However, to the best of our knowledge, no studies have investigated the incorporation of blueberry leaves into solid foods, representing an unexplored area and a prominent research gap in the development of functional foods.

Evidence from other species has demonstrated the feasibility of incorporating foliar materials, such as plant leaves or leaf-derived extracts, into bakery products to enhance functional properties. For instance, spent tea leaf powder has been incorporated into gluten-free shortbread cookies, improving antioxidant activity, fiber content, and sensory quality [14], while grape leaf phenolic extracts have been added to biscuits, retaining antioxidant properties during baking [15]. Enzymatically treated spent green tea leaf powder has also been used to produce high-fiber cookies, influencing dough texture, fiber composition, and overall acceptability [16]. Other studies have enhanced the nutritional properties of cookies using fruit pomace, vegetable flours, or other fiber-rich ingredients. Hence, cookies appear as a highly suitable platform among baked goods for nutritional and functional enrichment due to their widespread consumption, long shelf life, versatile formulations, and high consumer acceptability [17,18,19]. Therefore, due to the benefits of blueberry leaves, their direct incorporation into a cookie is an effective approach to addressing common dietary gaps such as insufficient fiber intake, low antioxidant consumption, and inadequate intake of protein and minerals. Moreover, blueberry-derived antioxidants may help neutralize oxidative stress and prevent cellular damage associated with aging and chronic diseases [20]. Adequate antioxidant intake is especially important in older adults for supporting cardiovascular and neurological health, as oxidative damage plays a significant role in the progression of age-related disorders such as heart disease and cognitive decline. Similarly, sufficient antioxidant intake in children is critical in contexts of poor dietary quality, obesity, or exposure to environmental stress, where oxidative damage may contribute to the development of early-onset metabolic or inflammatory conditions. Therefore, incorporating antioxidant-rich ingredients into accessible food formats, such as cookies, represents a practical and impactful public health strategy, particularly for reaching sensitive or at-risk populations [21].

Traditional cookies, however, are typically made with refined flour, sugar, and saturated fats, limiting their health benefits. This study also addressed this gap by incorporating BBLP into a reformulated cookie matrix based on a wheat and soy flour blend, aiming to develop a proof-of-concept product enriched with multiple protein sources, fiber, antioxidants, and calcium, thereby improving overall nutritional value without compromising sensory acceptance. Such nutrient-dense snacks offer promising options for populations with specific dietary needs and support the development of more balanced snacks tailored to vulnerable groups in low-income countries, where nutrient deficiencies are prevalent.

## 2. Results and Discussion

### 2.1. BBLP Characterization

The chemical characterization of BBLP revealed a compelling nutritional and functional profile (Table 1), composed of 7.2% moisture, 4.8% ash, 8.2% protein, and 44% total dietary fiber with 39% insoluble and 5% soluble fractions, positioning it as a moderately protein-rich and fiber-dense raw material. Biel and Jaroszewska [16] analyzed the nutritional value of leaves from selected berry species and reported the presence of protein, minerals, and crude fiber, supporting the potential of these leaves as promising ingredients for nutrient-enriched products. However, to date, no comprehensive proximate composition, including detailed dietary fiber fractions, has been reported specifically for blueberry leaves. Thus, this study provides a thorough nutritional profile of BBLP, which is critical for its valorization and application in functional food development.

The antioxidant properties of blueberry leaves have been typically investigated through solvent extraction of phenolic compounds. In this work, the obtained BBLP extract exhibited a total polyphenol content (TPC) and a total antioxidant capacity (TAC) of 2109 ± 20 mg GAE/100 g and 6251 ± 42 µmol TE/100 g, respectively (Table 1). These findings are consistent with previous reports on the phenolic profiles of blueberry leaves from different cultivars [6,8,13,22,23], which highlight their potential as sustainable sources of polyphenols and support their incorporation into antioxidant-enriched food formulations. However, the phenolic content in blueberry cultivars is influenced by a complex interplay of genetic, environmental, developmental, and methodological factors. The vegetative stage at which plant material was collected can markedly impact phenolic profiles, as these compounds fluctuate throughout plant development [23]. In addition, methodological differences in extraction and quantification (e.g., solvent selection, extraction procedures, calibration standards) can lead to variability in reported phenolic content across studies [22]. These factors must be carefully considered when evaluating or comparing the phenolic composition of blueberry leaves and other plant tissues. Nonetheless, our results confirm the high antioxidant potential of BBLP, particularly when the whole leaf matrix is preserved in food-grade powder form.

Free and bound phenolic compounds, with the latter covalently linked to plant cell wall structures, play distinct but complementary roles in human health. Free phenolics are typically absorbed in the upper gastrointestinal tract and contribute to systemic antioxidant effects. In contrast, bound phenolics resist early digestion and are gradually released through microbial fermentation in the colon, supporting gut health and exerting local antioxidant activity [24,25]. Using BBLP provides a valuable source of both bioactive and structural nutrients, as the whole leaf matrix retains not only soluble but also bound phenolic compounds. The latter are covalently or physically linked to plant cell wall components such as cellulose, hemicellulose, lignin, or pectin, and are not readily soluble in common extraction solvents [26,27]. By using whole dried leaves, both soluble (free) and cell wall-bound phenolics naturally occurring in the leaves are preserved in the final product. Moreover, the incorporation of BBLP into food products aligns with circular bioeconomy principles by reintegrating fruit production residues, such as powdered pruned leaves, into the food chain.

### 2.2. Cookies Characterization

#### 2.2.1. Chemical Composition

The proximate analysis of the obtained cookies formulated with increasing levels of BBLP showed that ash, total fat, saturated fatty acids (SFAs), unsaturated fatty acids (UFAs), trans fatty acids (TFAs), carbohydrates, calcium, and sodium contents remained statistically unchanged across all samples (Table 2). Noticeably, the calcium content in all formulations (≥1100 mg/100 g) qualifies them for “high in calcium” claims according to Codex Alimentarius guidelines and EU Regulation (EC) No 1924/2006 for foods [28]. On the other hand, the moisture content exhibited a slight but significant (*p* < 0.05) decrease at higher BBLP incorporation, ranging from 9.5% in the reference formulation (without BBLP) to 8.7% in the sample containing 7.5% BBLP. This reduction may be attributed to the higher insoluble fiber content introduced by BBLP, which can alter water-binding and retention properties during baking, leading to lower residual moisture in the final product [29,30]. The protein content also declined progressively with increasing BBLP levels due to the partial replacement of the flour mixture with the leaf powder, which contains a lower amount of proteins than the wheat and soy flours (BBLP: 10.5%; wheat and soy flours mixture: 47.2%, as determined experimentally in this study).

In contrast, the dietary fiber content of the BBLP-enriched cookies showed a marked nutritional improvement. In particular, the total dietary fiber (TDF) increased from 5.6% in the reference sample (i.e., 0% BBLP) to 7.8% in cookies with 7.5% BBLP, reflecting the fiber-rich nature of the leaf powder (Table 1). The incorporation of wheat flour, soy flour, and BBLP resulted in a composite fiber matrix comprising both soluble and insoluble fractions derived from different plant sources. Each ingredient contributes distinct fiber types: wheat flour primarily provides insoluble fiber, such as cellulose and arabinoxylans [31]; soy flour contributes a mixture of soluble and insoluble fibers, including pectins, oligosaccharides, and hemicelluloses [32]; and BBLP adds a substantial amount of insoluble fiber, particularly cellulose, hemicelluloses, and lignin—compounds typical of leaf tissue structure [33]. This compositional diversity may enhance the physiological functionality of the cookies by delivering fiber fractions associated with complementary health benefits, such as improved bowel regularity, modulation of glycemic response, and support for colonic fermentation and microbiota activity [34]. According to Codex Alimentarius guidelines (CAC/GL 23-1997; CAC/GL 2-1985) [35], products containing at least 3 g of fiber per 100 g (or 1.5 g per 100 kcal) may be labeled as a “source of fiber”. Additionally, foods with sodium content not exceeding 120 mg per 100 g qualify as “low in sodium”. These criteria align with the EU Regulation (EC) No 1924/2006 and serve as internationally recognized references for nutrition and health claims, often forming the basis for national labeling standards. In this context, the developed cookies meet the “source of fiber” claim based on their fiber content, qualify as “high in calcium” with calcium levels between 1108 and 1182 mg/100 g, and comply with the “low in sodium” criteria (96–103 mg/100 g), highlighting their potential as nutritionally enhanced products.

#### 2.2.2. Physical Analyses

The physical characteristics of the cookies formulated with increasing BBLP content are summarized in Table 3. Individual cookie weight remained statistically unchanged across all formulations (*p* > 0.05), ranging from 11.9 ± 0.2 to 13.5 ± 0.3 g. The partial replacement of the flour blend with BBLP did not significantly affect total dough mass or baking losses (i.e., weight loss primarily due to water evaporation). Cookie diameter and thickness decreased progressively with BBLP addition. This reduction can be attributed to the structural effect of dietary fiber, particularly insoluble fibers, which increase dough viscosity and firmness, thereby limiting its ability to flow and spread during baking. These findings align with the increased insoluble dietary fiber (IDF) content observed in the cookies as BBLP concentration rises (Table 1). Insoluble fibers act as physical fillers within the dough matrix, disrupting the gluten network, weakening dough structural integrity, and reducing its ability to retain gas bubbles produced during leavening [36]. Consequently, gas escapes more readily during baking, limiting dough expansion (smaller diameter) and resulting in thinner cookies. These dimensional changes were also reflected in the spread ratio, which increased significantly at 5.0% and 7.5% BBLP, indicating a dough matrix with reduced extensibility and weakened structure that spreads more laterally relative to thickness during baking due to increased fiber content. Cookie volume decreased accordingly, from 20 mL in the control formulation (0% BBLP) to 16–17 mL in the BBLP-enriched samples. This trend is consistent with lower gas retention typically observed in fiber-enriched doughs [36] and may also be linked to the lower moisture content reported in Table 2, which declined from 9.5% at 0% BBLP to 8.7% at 7.5% BBLP content, respectively. Reduced moisture likely contributed to decreased expansion and a more compact cookie structure during baking. Similar effects were reported by Chouaibi et al. [37], who observed that the addition of insoluble tomato fiber into wheat flour dough raised water absorption and increased the storage modulus (G’), resulting in higher dough viscosity. This led to decreased dough extensibility and a lower cookie spread ratio, indicating that the cookies retained their shape and spread less during baking. Moreover, Mancebo et al. [38] reported that sugar-snap cookies containing insoluble bamboo, potato, and pea fibers exhibited a lower spread factor and higher moisture content compared to control samples.

In terms of cookie texture, hardness increased significantly with BBLP concentration, from ~9 N in the sample with 0% BBLP to 13–14 N following the addition of 5.0% and 7.5% BBLP, suggesting a denser internal structure and reduced softness of the baked product. These results are consistent with the increase in insoluble fiber and the decrease in moisture retention observed in the cookies formulated with higher levels of BBLP (Table 1), both of which contribute to reduced tenderness. The observed trend is also consistent with other studies showing that the incorporation of insoluble fibers significantly increases cookie hardness. For instance, Mancebo et al. [38] reported that sugar-snap cookies containing insoluble fibers (such as bamboo, potato, and pea fibers) exhibited higher hardness compared to control samples. In addition, BBLP, added in powdered form, may act as rigid filler particles, reinforcing the cookie matrix, and increasing mechanical resistance. Furthermore, its presence may physically disrupt the continuity of the starch–protein network formed by wheat and soy flour during baking, compromising the structural cohesiveness and enhancing cookie firmness. A similar behavior was observed in a study where the incorporation of different commercial fibers (bamboo, cocoa, psyllium, chokeberry, and citrus) into a cookie recipe led to decreased dough spread and higher resistance to deformation, resulting in a firmer texture [39]. These effects are consistent with the rigid filler action of insoluble fibers, which can disrupt the starch–protein network and reinforce the cookie matrix.

**Figure 1:**
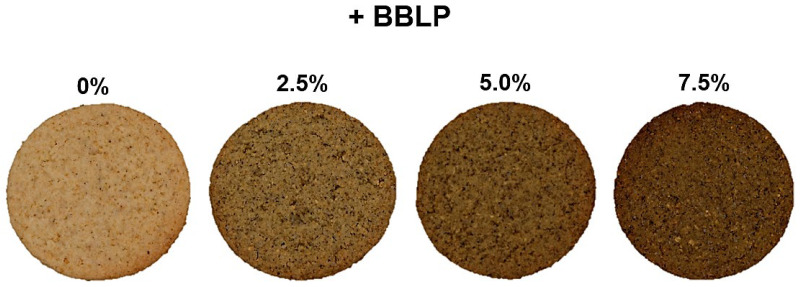
Representative images of cookies formulated with increasing content of BBLP.

Lastly, color evaluation confirmed that BBLP addition progressively darkened the cookies by a decrease in L* values (from 49 ± 1 at 0% BBLP to 25 ± 1 at 7.5% BBLP), along with increased redness (a*) and decreased yellowness (b*), resulting in a visual shift toward darker, reddish-purple hues (Figure 1). These changes are consistent with the presence of phenolic pigments, particularly red-purple anthocyanins, as well as chlorogenic acid and flavonoid derivatives, which are naturally abundant in blueberry leaves [40] and undergo transformation during baking, impacting color [41]. In addition, previous studies have reported that the incorporation of dietary fibers, particularly insoluble fibers, contributes to a darker cookie appearance. Mancebo et al. [38] observed that biscuits formulated with both soluble and insoluble fibers were darker than the control. Likewise, Gruppi et al. [39] demonstrated that the addition of bamboo, cocoa, psyllium, chokeberry, and citrus fibers to cookie dough reduced the lightness (L*) value and increased the a* parameter, resulting in darker and more reddish tones. Furthermore, cookies enriched with fibers or polyphenol- and anthocyanin-rich ingredients can undergo progressive darkening during baking, due to the intrinsic pigments of the functional ingredients and Maillard reactions. The latter generates melanoidins, brown-colored compounds that contribute to the overall color of the baked product [19,42,43].

#### 2.2.3. Antioxidant Activity

**Figure 2:**
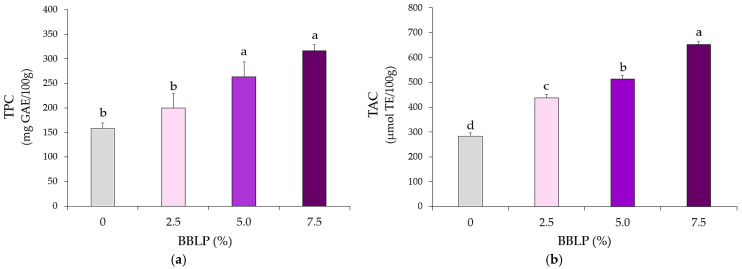
(**a**) Total polyphenol content (TPC) and (**b**) total antioxidant capacity (TAC) of cookies with increasing content of BBLP. GAE: gallic acid equivalent. TE: Trolox equivalent. Different letters indicate statistically significant differences (*p* < 0.05).

The total phenolic content (TPC) and total antioxidant capacity (TAC) of the cookies increased with the concentration of BBLP in the formulation (Figure 2). These results were expected due to the phenolic content and antioxidant activity of BBLP (Table 1). Despite the degradation of certain phenolic compounds during baking [41], the antioxidant capacity can be retained or even enhanced due to the formation of new antioxidant compounds. In fact, the Maillard reaction induced by baking generates melanoidins, which also possess antioxidant properties [19,42,43]. These compounds may act synergistically with the blueberry leaf-derived phenolics in the enriched cookies. For example, Kruczek et al. [44] demonstrated that gluten-free cookies supplemented with apple pomace retained high levels of phenolic acids, quercetin derivatives, flavan-3-ols, and dihydrochalcones after baking, attributing the sustained antioxidant activity to a combination of bound phenolics and Maillard reaction products. Similarly, Žilić et al. [19] found that cookies formulated with anthocyanin-rich corn flour maintained substantial antioxidant capacity post-baking, likely due to thermally induced antioxidant compounds. Consistent with these studies, our results demonstrate that the incorporation of BBLP, rich in both free and bound phenolics, enhances the antioxidant activity of cookies and preserves this activity after baking.

Despite growing scientific consensus on the protective role of polyphenols against chronic diseases such as cardiovascular and neurodegenerative disorders [20,21], current regulatory frameworks restrict antioxidant-related claims on food labels. As noted by Cory et al. [45], both the European Food Safety Authority (EFSA) and the U.S. Food and Drug Administration (FDA) require rigorous evidence of physiological benefits and proven bioavailability before approving health claims. In accordance with EU Regulation (EC) No 1924/2006, EFSA has rejected numerous generic “antioxidant” claims for polyphenols due to insufficient substantiation of a cause-and-effect relationship. Similarly, the FDA permits the use of the term “antioxidant” in labeling only for nutrients that have established Dietary Reference Intakes (DRIs) and recognized physiological antioxidant functions. These strict criteria highlight the gap between emerging scientific evidence and authorized consumer-facing claims.

#### 2.2.4. Sensory Analysis

**Figure 3:**
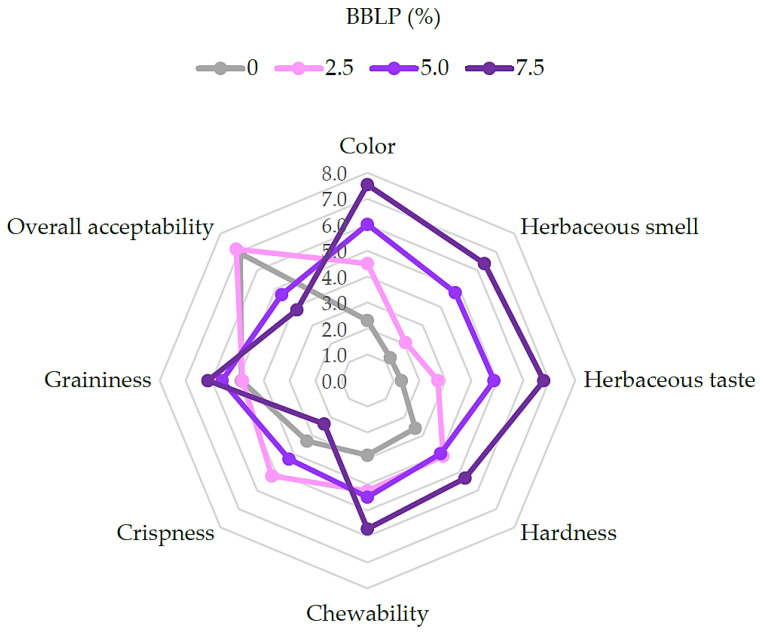
Spider plot showing the mean intensity scores for the sensory attributes of the cookies evaluated by QDA.

The Quantitative Descriptive Analysis (QDA) spider plot (Figure 3) illustrates how the sensory attributes of the cookies changed with the increasing levels of BBLP. Notably, color intensity, herbaceous aroma and flavor, hardness, and chewability (i.e., effort required for mastication) all increased with a higher BBLP content, significantly affecting appearance, texture, and taste across formulations.

Color progressively darkened (*p* < 0.05) with increasing BBLP levels, aligning with our previous results from Table 2 and Figure 1. Similarly, Žilić et al. [19] reported intensified browning in cookies enriched with anthocyanin-rich flours. This color change may be perceived positively by consumers, as it visually reinforces the presence of blueberry-derived compounds and the associated health benefits, potentially enhancing product appeal. In addition, the herbaceous smell and taste also intensified dose-dependently (*p* < 0.05), likely due to the presence of phenolic compounds such as chlorogenic acid and flavonol glycosides, which are known to contribute to bitter and astringent sensations in plant-based foods [46,47]. However, in the present study, these sensory attributes were not perceived as negative by the trained panel, suggesting that their intensity remained within an acceptable range and may even contribute to the natural character of the product. As sensory studies specifically focused on blueberry leaves are still scarce, this investigation represents a novel contribution to understanding the sensory implications of incorporating whole blueberry leaf powder into food matrices.

Texture showed a trend of increasing hardness and chewability with higher BBLP levels (0 < 2.5 = 5.0 < 7.5; *p* < 0.05), reaching maximum values at 7.5% BBLP. This indicates a denser and less aerated structure due to the increased fiber content, particularly insoluble fiber, as shown in Table 2 and discussed earlier in Section 2.2.1. The observed increase in graininess (0 = 2.5 < 5.0 < 7.5; *p* < 0.05) supports the presence of fibrous particles from the powdered leaves, which disrupt the typical smooth cookie crumb. In contrast, crispness significantly decreased (*p* < 0.05) as the BBLP content increased, likely due to the water-binding properties of insoluble fiber, which decrease moisture retention in the cookies after baking (Table 2) and also reduce air incorporation in the dough, resulting in a denser, less crisp texture of the final product. These effects are consistent with previous findings in fiber-enriched cookies and snacks [29,30]. Overall acceptability was highest for cookies with a moderate BBLP level—particularly at 2.5% incorporation—and comparable to the reference formulation (no BBLP added). Higher concentrations resulted in lower scores (*p* < 0.05), reflecting the balance between functional enrichment and sensory appeal, especially due to the increased bitterness and herbaceous aroma and taste.

**Figure 4:**
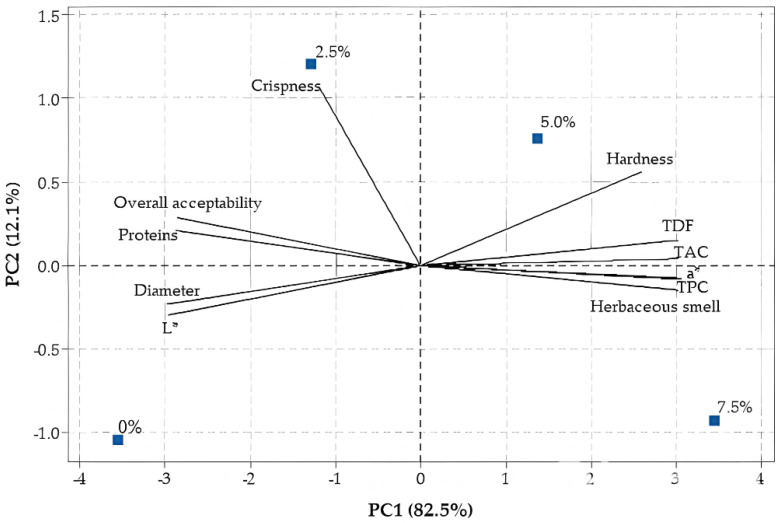
Principal Component Analysis (PCA) biplot of cookie samples with different percentages of BBLP addition. PC1 explains the majority of variance (85.2%) and separates samples based on BBLP level. Arrows represent the contribution of each property: positive correlations with BBLP include total phenolic content (TPC), total antioxidant capacity (TAC), hardness, total dietary fiber (TDF), redness (a*), and herbaceous smell; negative correlations include protein, diameter, crispness, lightness (L*), and overall acceptability. The 2.5% BBLP sample is positioned between low and high BBLP samples, indicating an optimal balance between functional enrichment and sensory acceptability.

In addition, a Principal Component Analysis (PCA) was conducted to explore multivariate relationships between cookie properties and BBLP addition, using representative nutritional (protein, TDF), physical/color (hardness, diameter, L*, a*), antioxidant (TPC, TAC), and sensory (crispness, herbaceous aroma, overall acceptability) attributes. The PCA biplot (Figure 4) shows a clear gradient along PC1, separating low (0–2.5%) from high (5.0–7.5%) BBLP-incorporated cookies. This indicates that BBLP introduction drives a consistent, multivariate shift in product attributes. Samples with higher BBLP levels (5.0–7.5%) clustered in the right portion of the plot, positively correlated with TPC, hardness, TDF content, darker color (a* redness), and herbaceous aroma, whereas cookies with low or no BBLP (0–2.5%) were located on the left, associated with higher protein, diameter, lightness (L*), crispness, and overall acceptability. Although PC1 explained most of the variance (85.2%), PC2 (12.1%) highlighted the unique positioning of moderate BBLP incorporation by further distinguishing the 2.5% BBLP cookies, which clustered higher along this axis and were strongly associated with crispness. Notably, the cookies with 2.5% BBLP also occupied an intermediate position along PC1, balancing enhanced nutritional properties and functional improvements with sensory quality. These results indicate that moderate BBLP incorporation (2.5%) provides functional benefits without strongly compromising consumer appeal.

### 2.3. Limitations and Future Perspectives

Some limitations of this study should be acknowledged. The antioxidant activity, phenolic content, and fiber were assessed under in vitro conditions, which may not fully reflect their bioavailability in vivo. As a proof-of-concept study, assessing the bioavailability of BBLP compounds, as well as calcium and protein, was not the primary focus; however, future works should address these aspects once an acceptable cookie formulation has been established under typical consumption conditions. In addition, a more detailed characterization of the specific antioxidant compounds and dietary fibers present in BBLP would help clarify their contribution to the functional and nutritional effects observed in the cookies at a molecular level. Further research should also examine the long-term stability and potential interactions of bioactive compounds with other macro- and micronutrients during cookie storage and digestion, as these factors can influence both antioxidant retention and in vivo bioavailability.

Regarding sensory evaluation, although this study followed established descriptive sensory methods and provides a solid first step in product characterization, larger consumer-based studies are needed to better capture population-level preferences and acceptance. Finally, rheological studies of the dough could offer valuable mechanistic insights into the physicochemical modifications observed in BBLP-enriched cookies. Taken together, these insights provide guidance for future studies aimed at fully realizing the functional potential of incorporating blueberry leaves—an underutilized agricultural by-product—into baked products.

## 3. Materials and Methods

### 3.1. Ingredients

Refined white wheat flour (Molino Juan Semino S.A., Santa Fe, Argentina), defatted soy flour (Hexal S.R.L., Máximo Paz, Buenos Aires, Argentina), trans-fat-free margarine (Compañía Argentina de Levaduras S.A.I.C., Lanús, Buenos Aires, Argentina), egg powder (Industria del Huevo, Rosario, Santa Fe, Argentina), baking powder (Kraft Foods Argentina, General Pacheco, Buenos Aires, Argentina), vanilla extract (La Virginia, Rosario, Santa Fe, Argentina), sugar (David Rosental & Hijos S.A.I.C., Rosario, Santa Fe, Argentina), dried blueberry leaves (*Vaccinium* spp.; “The Berry Store”, Rosario, Santa Fe, Argentina), calcium carbonate (Novalquim, Rosario, Santa Fe, Argentina), and water were used to prepare the cookie formulations. Notably, “The Berry Store” sources blueberry leaves from a commercial farm located in San Pedro (Buenos Aires, Argentina) where they are obtained as part of routine pruning. These leaves constitute a genuine underutilized agricultural by-product with potential for valorization in food applications.

### 3.2. BBLP Characterization

#### 3.2.1. Chemical Analyses

Blueberry leaves were milled with a commercial mill (Connoisserve, Lincoln, Buenos Aires, Argentina) and then successively sieved with a conventional manual sieve to obtain BBLP. The powder was stored in Ziploc bags until further analysis. Ash, protein, fat, moisture, and fiber contents of BBLP were determined according to AOAC methods (1998) [48]. Carbohydrate content was calculated by difference. Moisture content of BBLP was determined by drying 5 g samples at 105 °C to constant weight, following AOAC method 925.10 (Dalvo, Rosario, Santa Fe, Argentina). Protein content was calculated from nitrogen content determined by the Kjeldahl method (AOAC 979.09) using a conversion factor of 6.25. Fat content was determined by weighing after Soxhlet extraction of 5 g dried sample using a 50:50 mixture of petroleum ether and diethyl ether, following AOAC method 930.09. Ash content was determined by incinerating samples at 550 °C in a muffle furnace (ORL, Lomas de Zamora, Buenos Aires, Argentina) following AOAC method 923.03. Total dietary fiber was measured using the Megazyme Dietary Fiber Assay Kit (K-TDFR) according to AOAC method 991.43 (2000) [49]. All analyses were performed in triplicate (*n* = 3).

#### 3.2.2. Polyphenol Extraction and Antioxidant Assays

BBLP was subjected to chemical extraction following the procedure outlined by Žilić et al. [19], with modifications. Briefly, 0.5 of BBLP was digested in 4 M NaOH for 24 h at 25 °C. The mixture was then acidified to pH 2.0 using HCl and extracted four times with diethyl ether. After centrifugation, the combined ether fractions were evaporated to dryness, and the resulting residue was reconstituted in methanol. The total polyphenol content (TPC) was determined using the Folin–Ciocalteu colorimetric method [50]. For this, the methanolic extract was mixed with the Folin–Ciocalteu reagent, and the absorbance of the mixture was measured at 760 nm using a UV-Vis spectrophotometer (BIOTRAZA, CABA, Buenos Aires, Argentina). Gallic acid was used as the calibration standard. Results were expressed as milligrams of gallic acid equivalents per 100 g of sample (mg GAE/100 g). The total antioxidant capacity (TAC) was evaluated using the ABTS•+ radical cation decolorization assay, as described by Pukalskas et al. [51]. ABTS•+ was generated by reacting ABTS with 2.45 mmol/L potassium persulfate (K_2_S_2_O_8_) and allowing the mixture to stand in the dark for 16 h. The ABTS•+ solution was then diluted to an absorbance of 0.800 ± 0.030 at 734 nm, measured using the UV-Vis spectrophotometer. For the assay, 60 µL of the methanolic extract was mixed with 840 µL of the ABTS•+ solution, and the absorbance was recorded at 734 nm after 6 min. Trolox was used as the standard antioxidant. Results were expressed as millimoles of Trolox equivalents per 100 g of sample (mmol TE/100 g of sample). All assays were performed in triplicate (*n* = 3).

### 3.3. Experimental Protocol, Formulation, and Preparation of Cookies

A flour blend comprising 60% wheat and 40% soy flour was selected based on theoretical amino acid complementation to optimize lysine content, according to the amino acid profiles reported for whole-wheat flour and defatted soy flour by the USDA Food Data Central [52,53]. This blend was substituted with BBLP at 2.5%, 5.0%, and 7.5%. All other ingredients were kept constant, as detailed in Table 4. A reference cookie formulation without the functional ingredient (i.e., 0% BBLP) was also prepared and served as the control for evaluating the effects of BBLP incorporation.

The formulations were designed to deliver 35% of the recommended daily allowance (RDA) for calcium (1000 mg Ca^2+^/day for a healthy adult) in a 30 g portion (~3 cookies) [35] (Annex 1). Cookie dough was prepared using a standard kitchen mixer (ATMA, Tierra del Fuego, Ushuaia, Argentina), following a sequential mixing process. Initially, a pre-mixed blend of margarine, sugar, and powdered egg was prepared using an electric mixer (Philips, CABA, Buenos Aires, Argentina). Next, the dry ingredients—flours, BBLP, calcium carbonate, and baking powder—were first homogenized and subsequently incorporated into the mixture alternately with water and vanilla extract. The dough was refrigerated for 40 min to achieve optimal consistency for lamination, then rolled (Sol Real, Rosario, Santa Fe, Argentina), cut into 5 cm circular shapes, and baked in a convective electric oven (ZONDA, Pilar, Buenos Aires, Argentina) at 180 °C for 8 min. After baking, cookies were cooled at room temperature and packaged in Ziploc bags. Each cookie formulation was produced in two independent replicates under identical baking conditions and subsequently evaluated using the same methodologies, ensuring consistency, reliability, and fair comparison of physicochemical properties and sensory attributes across samples.

### 3.4. Characterization of Cookies

#### 3.4.1. Chemical Analyses

The chemical composition of the cookies was analyzed in ground cookies according to the methodologies detailed in Section 3.2.1. Furthermore, the fatty acid profile of the cookies was analyzed based on the total fat content using HPLC-MS (Agilent 7890B, Agilent Technologies, Santa Clara, CA, USA) to determine the proportions of saturated fatty acids (SFAs), unsaturated fatty acids (UFAs), and trans fatty acids (TFAs). Calcium content was quantified by flame atomic absorption spectrometry using a SOLAAR 969 instrument (UNICAM, Cambridge, UK), following AOAC Method 985.35 [49]. Samples were digested with concentrated nitric acid, diluted appropriately, and analyzed with strontium nitrate added as a releasing agent to eliminate potential phosphate interference during atomization. Sodium content was determined by flame emission spectrophotometry using a WAYERS 2000 instrument (CABA, Buenos Aires, Argentina), following a modified AOAC Method 984.27 [49]. Samples were incinerated in a muffle furnace to obtain mineral ash, which was then dissolved in 4 N HCl for quantification against reference standards.

#### 3.4.2. Physical Analyses

Physical characteristics of the cookies, including weight, volume, diameter, thickness, spread factor, texture, and color, were evaluated. Five cookies per batch were individually weighed using an analytical balance with a precision of 0.1 g. Diameter was measured by placing six cookies edge to edge, then rotating the arrangement 90 degrees, and averaging the measurements [17]. Thickness was measured by stacking the same six cookies in different orders and recording the height using a caliper. The spread ratio was calculated by dividing the average diameter by the average thickness, as described by Abdel-Moemin [40]. All these measurements were performed in triplicate (*n* = 3).

Texture was evaluated using a three-point bending test, performed with a motorized testing frame (Multitest 2.5-d, Mecmesin, Slinfold, UK) equipped with a triple beam apparatus. Each cookie was positioned on two support anvils, and a compression bar descended at a rate of 180 mm/min until fracture occurred. Hardness, defined as the force required to break the cookie, was obtained from the peak of the force–deformation curve [18]. Measurements were performed in quintuplicate (*n* = 5).

Color was evaluated on the top surface of the cookies using digital image analysis. A standardized light box equipped with D65 fluorescent lamps (6504 K, Biolux 18W/965, Osram, Munich, Germany) was used to ensure consistent illumination during image capture. Images were taken with a Canon EOS Rebel T3 (Tokio, Japan) (ISO 400, 1/200 s, no flash) positioned 30 cm above the sample. A calibration card (IT8, Wolf Faust, Germany) and LProf software (version 1.11.2) were used for profiling. Color coordinates (L, a, b) were extracted using Adobe Photoshop and converted to CIE Lab parameters, following the method of Yam and Papadakis [54]. The assay was performed in quintuplicate (*n* = 5).

#### 3.4.3. Antioxidant Capacity

Chemical extracts of ground cookies were prepared following the procedure outlined by Žilić et al. [19], as detailed in Section 3.2.2. These extracts were used to determine TPC (mg GAE/100 g of sample) and TAC (µmol TE/100 g of sample), as previously described. All assays were performed in triplicate (*n* = 3).

#### 3.4.4. Descriptive Sensory Analysis

A descriptive sensory profiling was conducted by a trained panel of eight members composed of four males and four females (aged 22–54), following Stone et al. [55]. The non-smoking panelists were specifically selected and trained to evaluate baked products. Recruitment took place at the Faculty of Biochemical and Pharmaceutical Sciences, National University of Rosario (Santa Fe, Argentina). Training was successfully completed after several screening assays, which included aroma recognition, basic taste identification, discrimination using the triangle test, and descriptive evaluations of various baked products, such as different types of bread and cookies [55]. Based on consensus descriptors (Table 5), a finalized sensory evaluation form was developed using a 10 cm unstructured line scale. Each scale was anchored 1 cm from both ends to indicate minimum and maximum intensity of each attribute, progressing from left to right (e.g., from “weak” to “strong” or “light” to “dark”). To ensure consistency in interpretation and scoring, each descriptor was accompanied by a precise definition established during the training phase, which consisted of two sessions per week over one month.

The evaluated sensory attributes included color intensity, herbaceous odor and flavor, hardness (force required for the first bite), chewability (number of mastication cycles prior to swallowing), crispness (degree of fracturing perceived during chewing), graininess (perception of particles between the tongue and palate), and overall quality, defined as the harmonious integration of all evaluated attributes [56]. Each evaluation booth was equipped with an evaluation sheet, a pen, a glass of water, a disposable napkin, and reference materials. Once all panelists were seated in their assigned booths, four samples of each cookie were distributed on white trays. The samples were presented simultaneously to all panelists following a predetermined order and were labeled with randomly assigned three-digit codes. This approach ensured that each panelist received the same samples in the same sequence and under identical coding conditions, thereby promoting consistency throughout the evaluation process. Panelists’ responses were quantified by measuring the distance (in cm) from the left anchor of the scale.

### 3.5. Statistical Analysis

Data analysis was carried out using the Statgraphics Plus 5.1 program (Statpoint Technologies, Warrenton, VA, USA). One-way analysis of variance (ANOVA) was used to assess differences among groups. When significant differences were detected (*p* < 0.05), Tukey’s Honestly Significant Difference (HSD) test was applied for post hoc comparisons. For the sensory evaluation, ANOVA was also used to evaluate the effect of BBLP addition on the different sensory attributes and to examine panelist consistency for each sample, while triangle tests were performed to assess panel discrimination ability, thereby supporting the validation of the sensory profile. PCA was conducted to explore multivariate relationships between cookie properties across BBLP substitution levels (0–7.5%). Data included standardized variables of nutritional, physical, antioxidant, and sensory attributes. PCA was performed in Minitab^®^ Statistical Software, version 19.1 (Minitab LLC, State College, PA, USA) and results were visualized as a two-dimensional biplot.

## 4. Conclusions

The incorporation of blueberry leaf powder (BBLP) into cookie formulations was investigated as a strategy to enhance the nutritional and functional properties of baked products. Unlike previous studies that focused primarily on phenolic extracts, this work utilized the whole leaf matrix, representing an innovative and sustainable use of a traditionally discarded agricultural by-product. The addition of BBLP resulted in significant increases in total phenolic content and antioxidant capacity, reflecting the phytochemical richness of the whole leaf. Additionally, cookies enriched with BBLP showed a higher dietary fiber content and notable changes in color and texture, yet these modifications did not pose major technological challenges. While higher BBLP levels intensified herbaceous flavor, hardness, and graininess, overall acceptability remained within acceptable limits, particularly for cookies containing 2.5% BBLP, which achieved a favorable balance between nutritional enhancement and sensory quality. These findings underscore the potential of BBLP as a functional ingredient for bakery applications and demonstrate the feasibility of incorporating whole leaf material into food matrices. Future studies should investigate the shelf life and bioavailability of bioactive compounds in BBLP-enriched products, evaluate consumer acceptance at a larger scale, and explore the applicability of BBLP in other food categories to support the development of clean-label products and potential health impact. Furthermore, expanding the use of BBLP can contribute to sustainable food production by valorizing agricultural waste streams, thus supporting circular economy principles in the food industry.

## Figures and Tables

**Table 1:** Characterization of BBLP.

Component	Content
Moisture (% W/W)	7.2 ± 0.4
Ashes (% W/W)	4.8 ± 0.1
Proteins (% W/W)	8.2 ± 0.1
Fat (% W/W)	2.2 ± 0.1
TDF (% W/W)	44 ± 1
IDF (% W/W)	39 ± 1
SDF (% W/W)	5 ± 2
TPC (mg GAE/100 g)	2109 ± 20
TAC (µmol Trolox/100 g)	6251 ± 42

BBLP: blueberry leaf powder. TDF: total dietary fiber; IDF: insoluble dietary fiber; SDF: soluble dietary fiber. TPC: total polyphenol content. TAC: total antioxidant capacity.

**Table 2:** Chemical characterization of cookies with increasing content of BBLP (g/100 g of cookies).

Component	BBLP (%)			
	0	2.5	5.0	7.5
Moisture	9.5 ± 0.1 ^a^	9.3 ± 0.08 ^ab^	9.2 ± 0.1 ^b^	8.7 ± 0.1 ^c^
Ash	3.7 ± 0.1 ^a^	3.6 ± 0.1 ^a^	3.7 ± 0.2 ^a^	3.6 ± 0.1 ^a^
Proteins	12.0 ± 0.1 ^a^	11.6 ± 0.1 ^b^	11.5 ± 0.1 ^b^	10.7 ± 0.1 ^c^
Fats	21.5 ± 0.4 ^a^	21.2 ± 0.1 ^a^	21.7 ± 0.2 ^a^	21.2 ± 0.4 ^a^
SFAs	11.2 ± 0.3 ^a^	10.3 ± 0.1 ^a^	10.7 ± 0.1 ^a^	10.3 ± 0.1 ^a^
UFAs	10.3 ± 0.3 ^a^	10.9 ± 0.1 ^a^	11.0 ± 0.1 ^a^	10.9 ± 0.1 ^a^
TFAs	0.40 ± 0.01 ^a^	0.39 ± 0.02 ^a^	0.40 ± 0.02 ^a^	0.40 ± 0.01 ^a^
TDF	5.6 ± 0.4 ^b^	6.5 ± 0.5 ^ab^	7.2 ± 0.5 ^ab^	7.8 ± 0.4 ^a^
IDF	3.4 ± 0.4 ^b^	4.5 ± 0.5 ^ab^	5.1 ± 0.6 ^ab^	5.6 ± 0.1 ^a^
SDF	2.2 ± 0.1 ^a^	2.0 ± 0.4 ^a^	2.1 ± 0.4 ^a^	2.2 ± 0.5 ^a^
Carbohydrates	47.9 ± 0.5 ^a^	48.0 ± 0.4 ^a^	46.6 ± 0.6 ^a^	48.0 ± 0.4 ^a^
Calcium *	1182 ± 141 ^a^	1174 ± 40 ^a^	1162 ± 81 ^a^	1108 ± 42 ^a^
Sodium *	96 ± 7 ^a^	97 ± 5 ^a^	103 ± 6 ^a^	102 ± 3 ^a^

SFAs: saturated fatty acids; UFAs: unsaturated fatty acids; TFAs: trans fatty acids; TDF: total dietary fiber; IDF: insoluble dietary fiber; SDF: soluble dietary fiber. Values with different letters in each line are significantly different (*p* < 0.05). * Values expressed in mg/100 g of cookies.

**Table 3:** Physical parameters of cookies with increasing content of BBLP.

Parameter	BBLP (%)			
	0	2.5	5.0	7.5
Individual weight (g)	13.0 ± 1.0 ^a^	13.5 ± 0.3 ^a^	12 ± 1.0 ^a^	11.9 ± 0.2 ^a^
Diameter (cm)	5.30 ± 0.02 ^a^	5.14 ± 0.03 ^b^	5.09 ± 0.05 ^b^	5.01 ± 0.02 ^bc^
Thickness (cm)	0.90 ± 0.01 ^a^	0.86 ± 0.01 ^b^	0.82 ± 0.01 ^c^	0.81 ± 0.01 ^c^
Spread Ratio	5.8 ± 0.1 ^b^	6.0 ± 0.1 ^b^	6.20 ± 0.03 ^a^	6.15 ± 0.03 ^a^
Volume (mL)	20 ± 2 ^a^	18 ± 1 ^b^	16 ± 1 ^bc^	17 ± 1 ^bc^
Hardness (N)	9 ± 1 ^b^	12 ± 1 ^ab^	14 ± 2 ^a^	13 ± 2 ^a^
L*	49 ± 1 ^a^	36 ± 1 ^b^	28 ± 1 ^c^	25 ± 1 ^d^
a*	8.2 ± 0.5 ^b^	8.6 ± 0.3 ^b^	9.6 ± 0.5 ^a^	10.1 ± 0.4 ^a^
b*	25 ± 1 ^a^	24.6 ± 0.3 ^b^	23.6 ± 0.4 ^c^	22.1 ± 0.3 ^d^
BI	82 ± 4 ^d^	124 ± 7 ^c^	170 ± 8 ^b^	191 ± 10 ^a^

Diameter and thickness were repeated with three groups of six cookies. Volume, hardness, and color were performed in quintuplicate. BI: brownness index. Results are expressed as mean values ± SD. In CIELAB, L* represents lightness, a* the green–red axis, and b* the blue–yellow axis, all of which are psychometrically scaled so that equal numerical differences (ΔE*) correspond, as closely as possible, to equal perceived differences in human vision. Values with different letters in each line are significantly different (*p* < 0.05).

**Table 4:** Recipes of different cookies.

Ingredients (% W/W)	BBLP (%)			
	0	2.5	5.0	7.5
Wheat flour	26.8	25.2	23.6	21.9
Soy flour	13.9	13.1	12.2	11.4
Margarine	20.4	20.4	20.4	20.4
Water	10.6	10.6	10.6	10.6
Egg powder	3.1	3.1	3.1	3.1
Baking powder	0.8	0.8	0.8	0.8
Vanilla extract	0.3	0.3	0.3	0.3
Sugar	20.4	20.4	20.4	20.4
Calcium carbonate	3.6	3.6	3.6	3.6
BBLP	0.0	2.5	5.0	7.5

BBLP: blueberry leaf powder.

**Table 5:** Descriptions, anchors, and references for the descriptive sensory analysis of the cookies.

Descriptor	References	
Color: color intensity	1 = light Reference: alfajor cookie layers FANTOCHE^®^	9 = dark Reference: cookie “Chocolinas” ARCOR^®^
Herbaceous smell: herbaceous notes intensity in smell	1 = light Reference: water	9 = very Reference: blueberry leaf powder
Herbaceous taste: herbaceous notes intensity in taste	1 = light Reference: water	9 = very Reference: blueberry leaf powder
Hardness: force applied at first bite, evaluated by front teeth	1 = light Reference: alfajor cookie layers FANTOCHE^®^	9 = very Reference: cookie “Muesli” MURKE^®^
Chewability: number of times that is necessary masticate to allow the deglutition	1 = light Reference: water	9 = very Reference: cookie “Muesli” MURKE^®^
Crispness: quantifies the food shattering in mouth	1 = light Reference: water	9 = very Reference: cookie “Muesli” MURKE^®^
Graininess: granules perception by pressing masticated food between tongue and palate	1 = light Reference: water	9 = very Reference: cookie “Muesli” MURKE^®^
Overall acceptability: harmony level of all mentioned parameters	1 = light	9 = very

## Data Availability

The data presented in this study are available on request from the corresponding author due to privacy reasons.
